# Supplementary material for: A universal deep neural network for in-depth cleaning of single-cell RNA-Seq data
Source: Nat Commun. 2022 Apr 7;13:1901. doi: 10.1038/s41467-022-29576-y (PMC8990021; doi:10.1038/s41467-022-29576-y)
Supplement: Supplementary file 1 — Supplementary Information New [file 41467_2022_29576_MOESM1_ESM.pdf]

A Universal Deep Neural Network for In-Depth Cleaning of Single-Cell RNA-Seq Data

Hui Li[1,2], Cory R. Brouwer[1,2], Weijun Luo[1,2,3*]

[1]Department of Bioinformatics and Genomics, College of Computing and Informatics, UNC Charlotte, Charlotte, NC 28223

[2]UNC Charlotte Bioinformatics Service Division, North Carolina Research Campus, Kannapolis, NC 28081

[3]Novant Health, Charlotte, NC 28207 (current address)

[*]Correspondence: luo_weijun@yahoo.com

Supplementary Materials

Supplementary Figures 1 to 11

Supplementary Tables 1 to 3

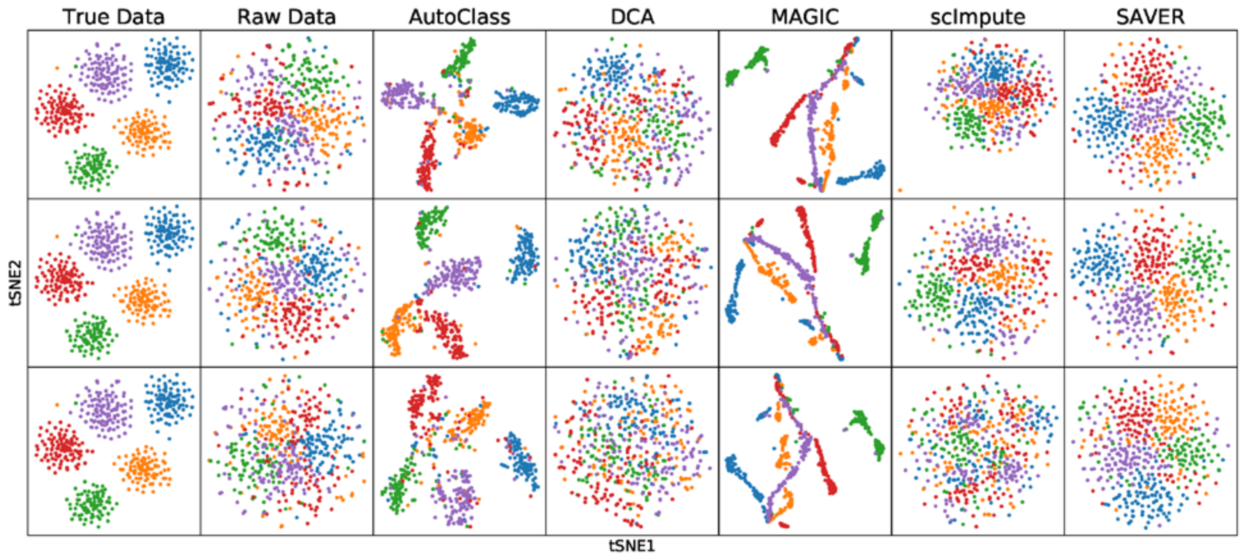

Supplementary Figure 1 t-SNE plots for Dataset 3 (first row, random uniform noise), Dataset 5 (second row, Gamma noise) and Dataset 6 (third row, Poisson noise). Experiments settings are similar to those in Figure 2.

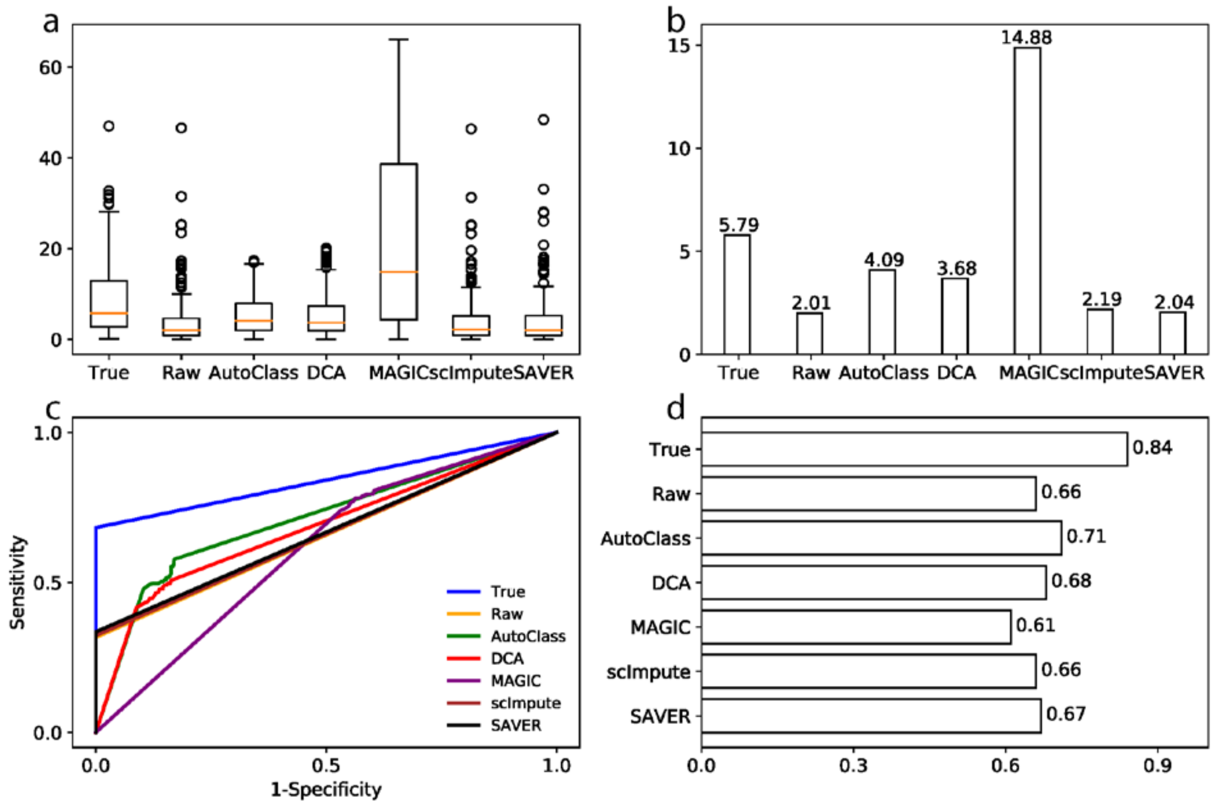

Supplementary Figure 2 Differential expression analysis for Dataset 9 (Gaussian noise). **a** and **b** T-statistics and their median for truly differentially expressed genes. **c** and **d** ROC curves and areas under the ROC curves. Experiments settings are similar to those in Figure 3 a-d. In **a**, the box represents the interquartile range, the horizontal line in the box is the median, and the whiskers represent 1.5 times the interquartile range, with sample size n=53 marker genes.

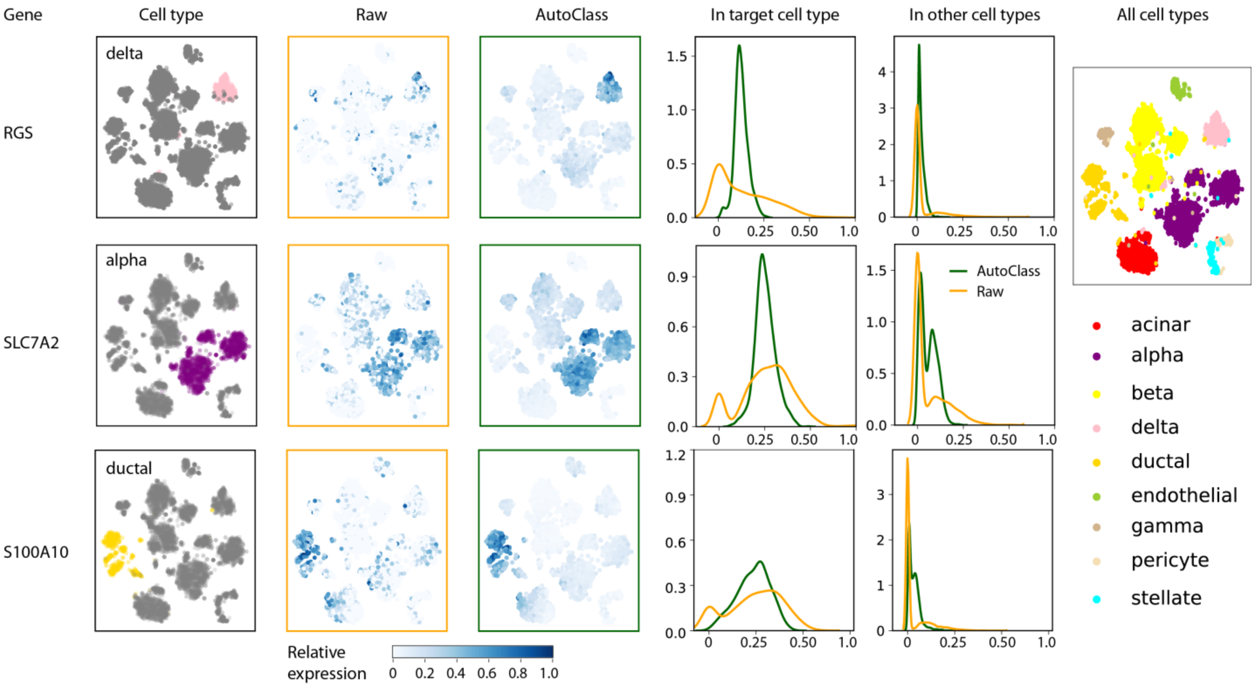

Supplementary Figure 3 AutoClass improves potential marker genes identification. a. RGS2 in delta cells, b SLC7A2 in alpha cells, c S100A10 in ductal cells. The expression data showed in column 2-5 is the relative log2 expression, i.e. log2 based expression scaled by the maximum of each gene in raw data. The t-SNE plots were generated from raw data.

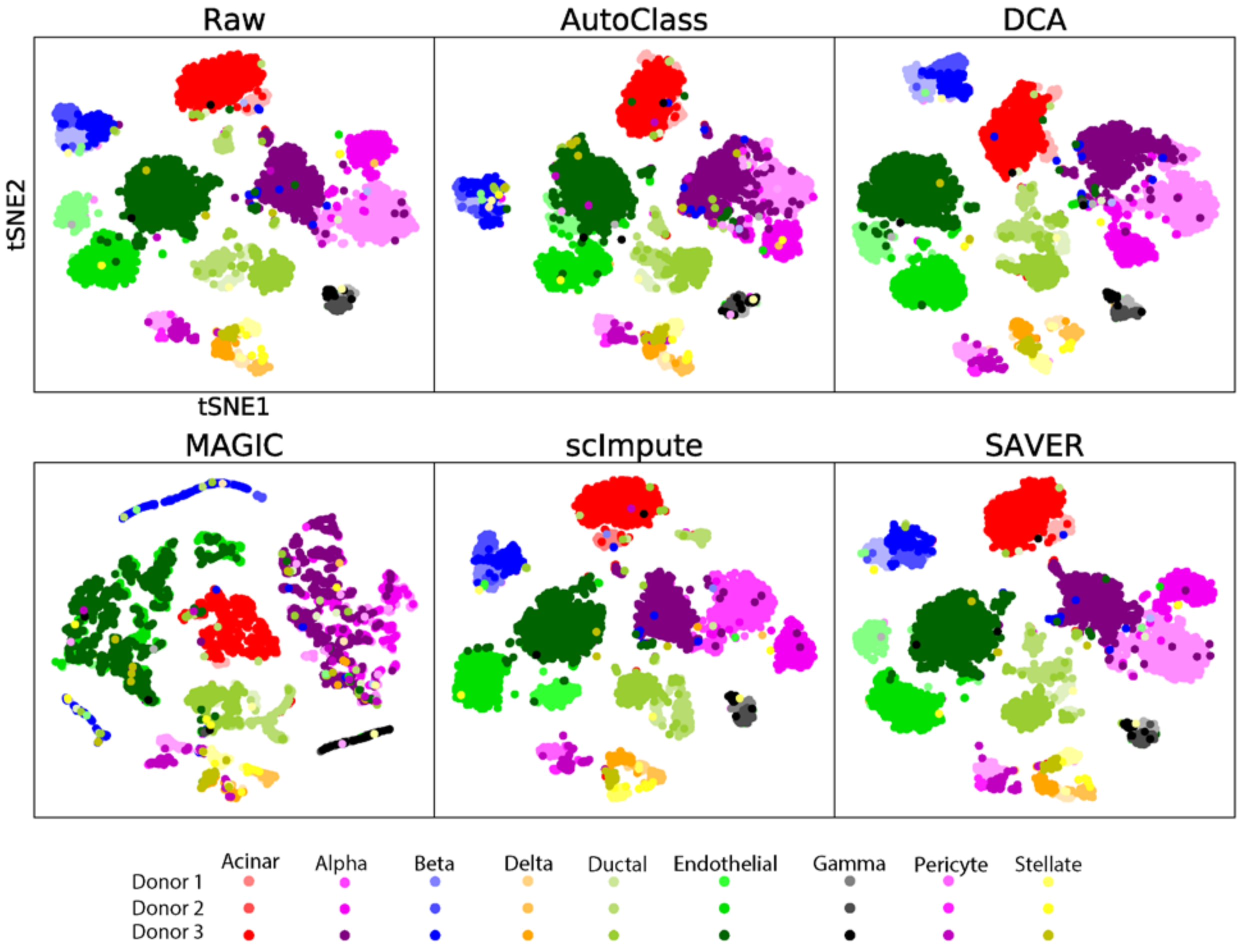

Supplementary Figure 4 t-SNE plots for raw and imputed data for Baron dataset. Experiments settings are similar to those in Figure 5a.

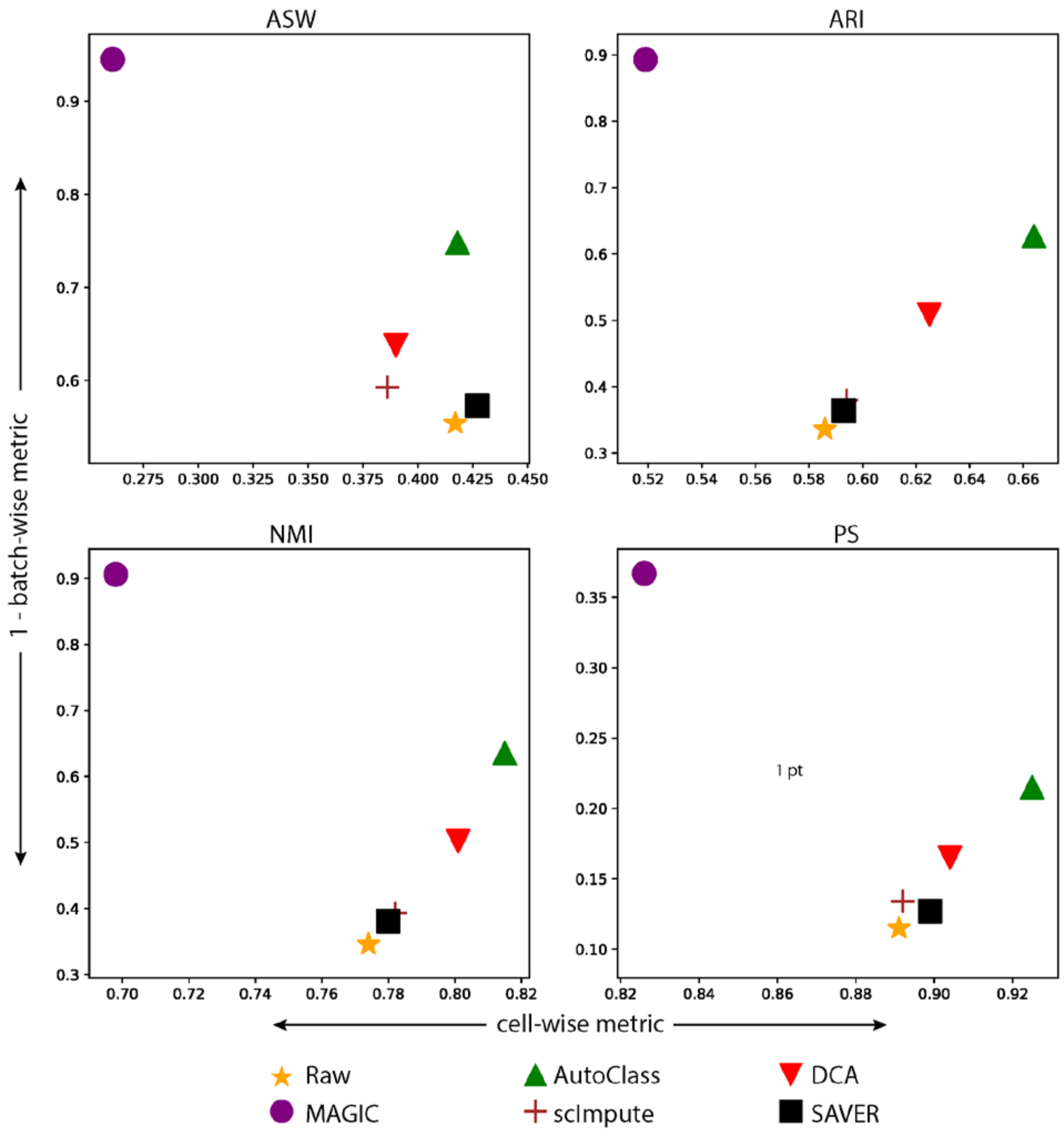

Supplementary Figure 5 Evaluation of batch and cell type separation in raw and imputed data by four different metrics for Baron dataset. Experiments settings are similar to those in Figure 5b.

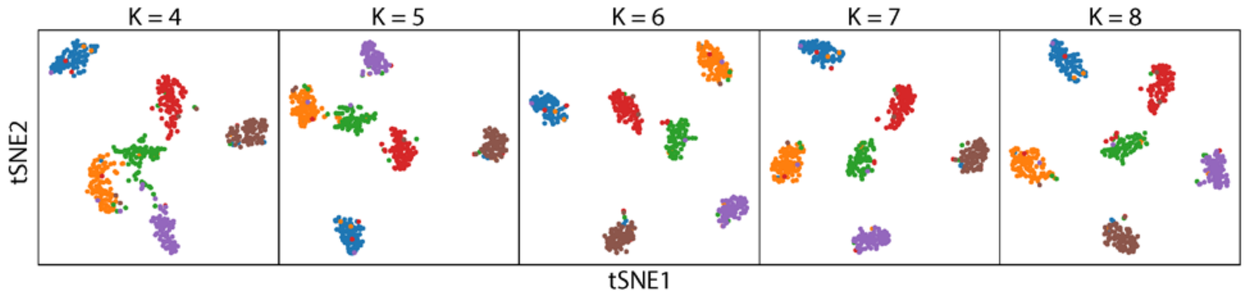

Supplementary Figure 6 t-SNE plots of Dataset 1 imputed by AutoClass with different K in pre-clustering. Experiments settings are similar to those in Figure 6a.

.

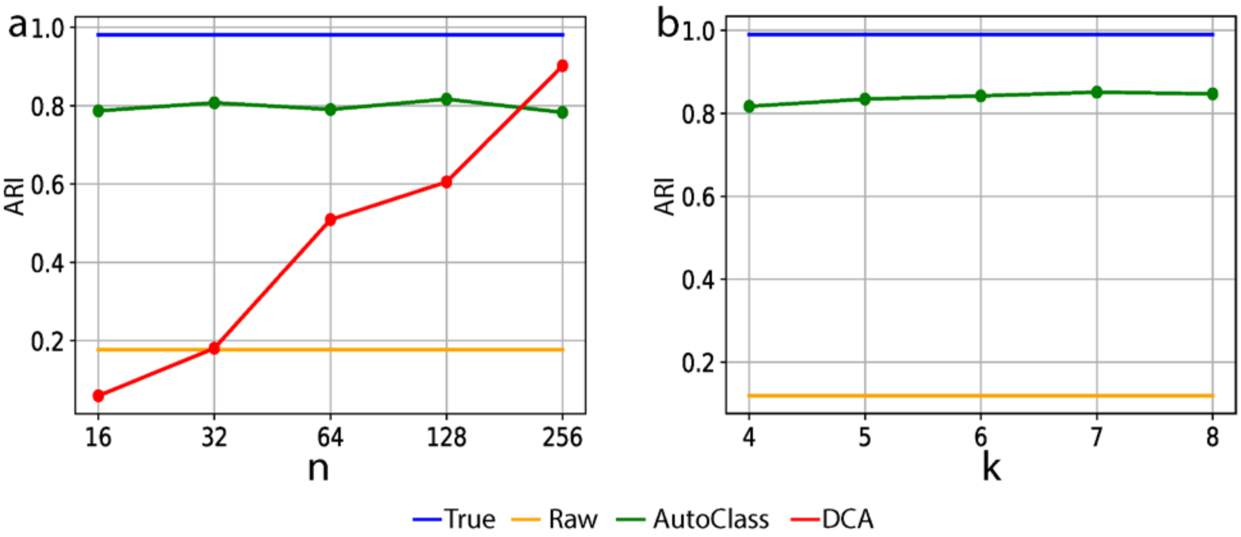

Supplementary Figure 7 ARI of K-means clustering on t-SNE transformed data for Dataset 1 over **a** different bottleneck layer sizes and **b** different choices of K in pre-clustering of AutoClass (details are described in main text and Methods). Experiments settings are similar to those in Figure 6c and 6d.

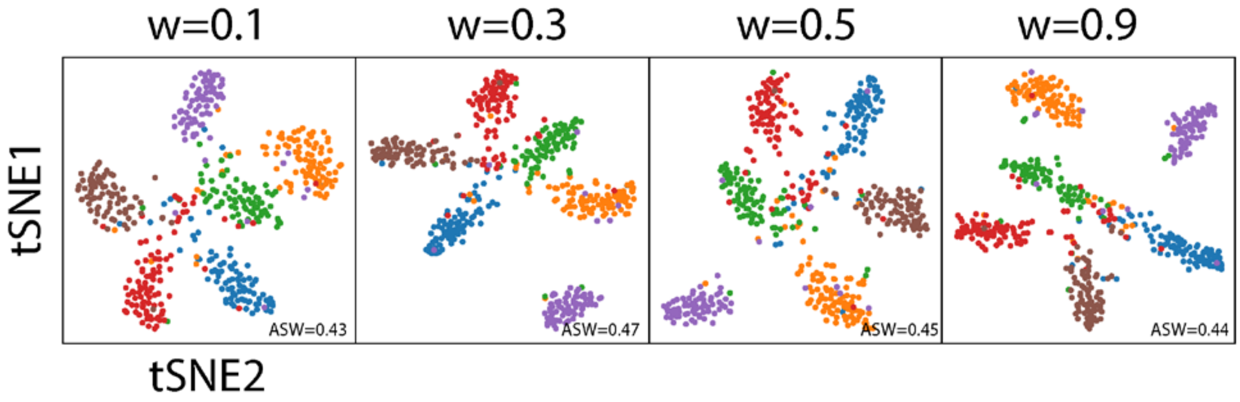

Supplementary Figure 8 t-SNE plots for Dataset 1 after AutoClass imputation over different classifier weights. Experiments settings are similar to those in Figure 6a.

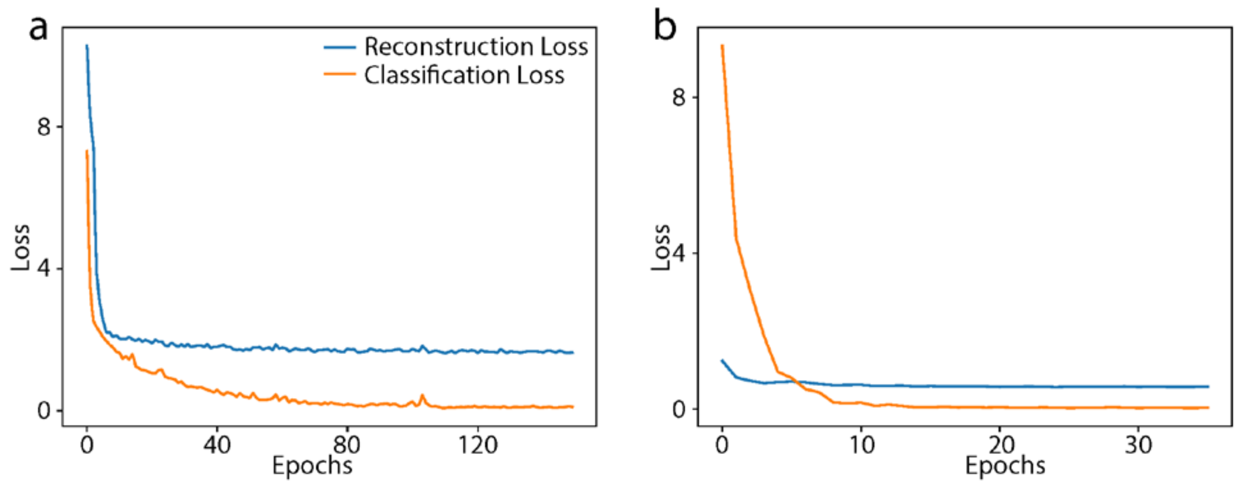

Supplementary Figure 9 AutoClass loss curves. **a** Dataset 1**. b** Buettner dataset.

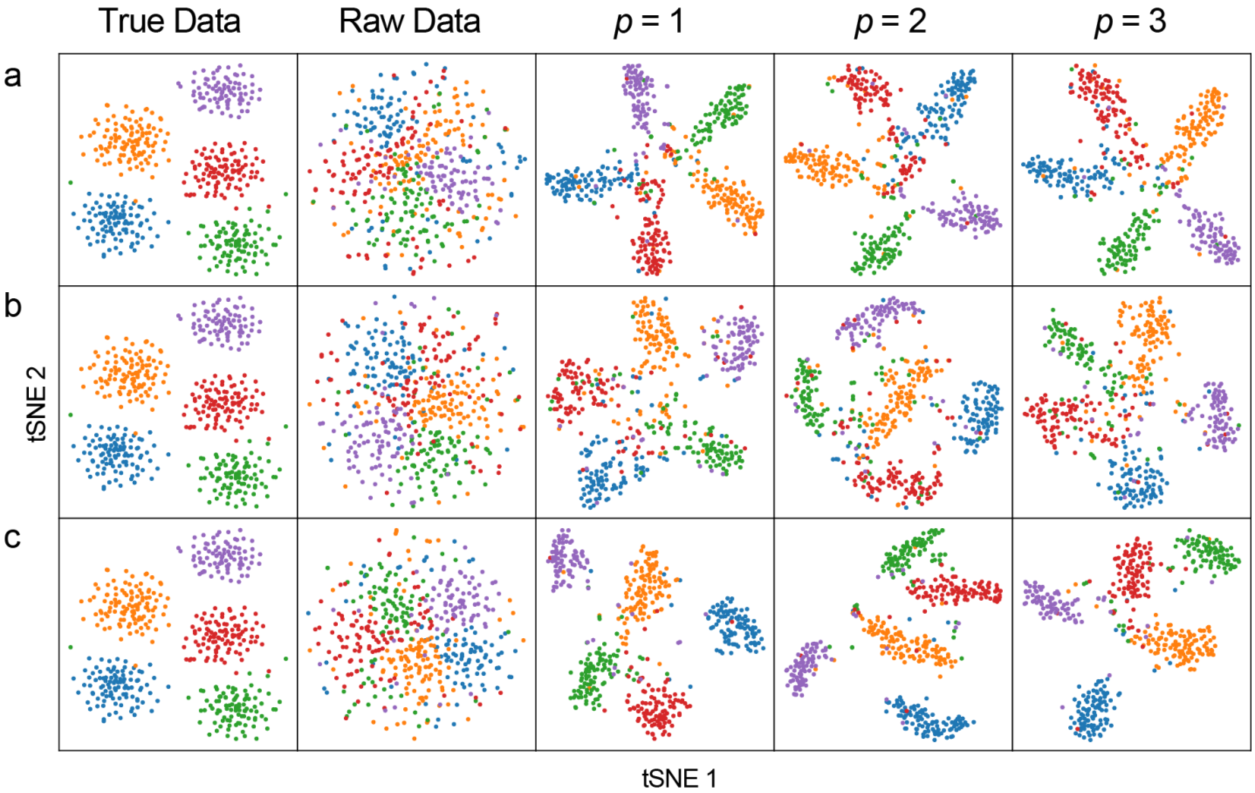

Supplementary Figure 10. Denoising using AutoClass with different p parameter values in its reconstruction loss $|\overline{X} - Y_k|^p$. **a** Dataset 2 (dropout noise), b Dataset3 (Uniform noise) and **c** Dataset 7 (Negative Binomial noise). Experiments settings are similar to those in Figure 6a.

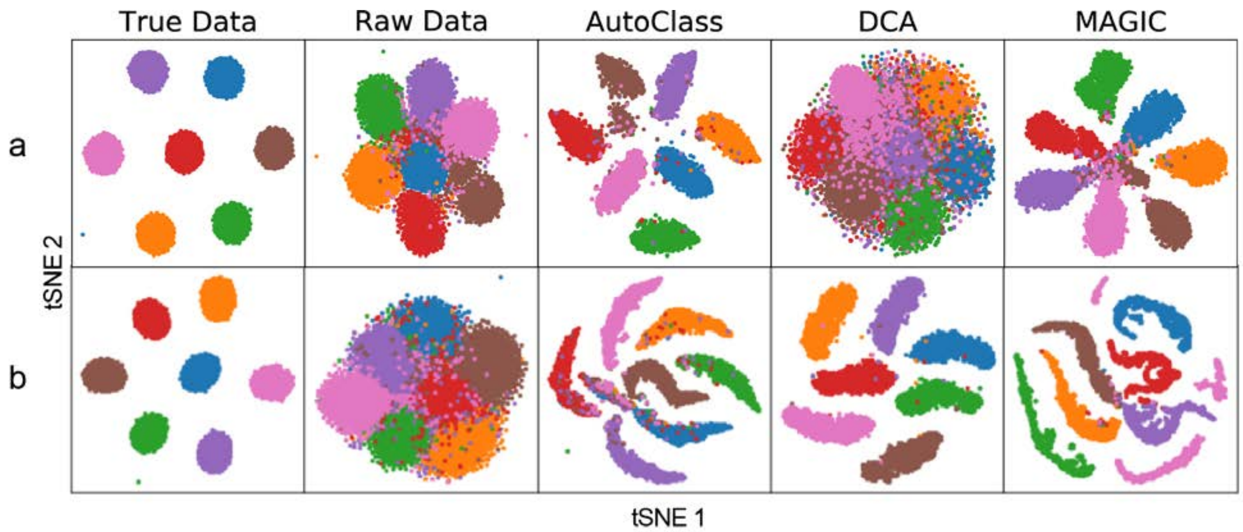

Supplementary Figure 11 Imputation results for simulated datasets with both large feature size (10000 genes) and sample size (10000 cells). Data were simulated using **a** Li et al Method[11] (Dataset 10), and **b** Splatter[13] (Dataset 11). Note SAVER and scImpute were included in these large datasets analyses because they are too slow (Figure 7) and could not complete these tasks in a reasonable amount of time on our working machine.

Dataset[Ref]	Description	Cell groups	Sample batches	Cells	% zero counts	AutoClass setting
Baron[16]	Human pancreatic islets cells	8	3	7,162	82.21	*dropout_rate=0.3*
Villani[26]	Human blood dendritic cells	4	2	768	47.33	*dropout_rate=0.3*
Lake[20]	Human brain frontal cortex cells	11	NA	8,592	73.39	Default
Zeisel[21]	Mouse cortex and hippocampus cells	9	NA	3,005	48.58	Default
Buettner[2]	Mouse embryonic stem cells	3	NA	182	37.92	Default
Usoskin[19]	Mouse neuronal cells	4	NA	622	95.83	Default
Tian[27]	Single cell mixture	5	NA	305	64.27	Default

Supplementary Table 1 Summary of real scRNA-seq datasets used in this study.

Dataset	Genes/Cells	Cell groups	Splatter dropout[*]	AutoClass setting
Dataset 1	1000/500	6	-1/5/1.5	Default
Dataset 2	1000/500	5	-2.5/2.5	Default
Dataset 8	1000/500	2	-1/3	*num_cluster* = [2,3,4]
Dataset 10	10000/10,000	7	NA[#]	Default
Dataset 11	10000/10,000	7	-1/2.5	Default
Scalability Datasets	1000/1000-32000	5	-2.5/2.5	Default
Tian	1000/305	5	-2.5/3.5	Default
Tian	5000/305	5	-2/3	Default

Supplementary Table 2 Summary for simulated scRNA-seq datasets with dropout noise used in this study.
[*]Splatter dropout settings: *dropout.shape/dropout.mid.* [#] *dropout added following Li et al method*[11].

Dataset	Noise Type	*numpy.random* function	numpy setting	AutoClass setting
Dataset 3	Uniform	*randint*	low=-10, high=10	Default
Dataset 4	Gaussian	*normal*	loc=0, scale=6	Default
Dataset 5	Gamma	*gamma*	shape=0.5, scale=12	Default
Dataset 6	Poisson	*poisson*	lam=6	Default
Dataset 7	Negative Binomial	*negative_binomial*	n=10, p=0.45	Default
Dataset 9	Gaussian	*normal*	loc=0, scale=18	*num_cluster* = [2,3,4]
Tian (1k genes)	Negative Binomial	*negative_binomial*	n=10, p=0.2	Default
Tian (5k genes)	Negative Binomial	*negative_binomia*	n=15, p=0.2	Default

Supplementary Table 3 Summary for simulated scRNA-seq datasets with non-dropout noises used in this study. Note Dataset 3-7 were generated from the true data of Dataset 2 above, Dataset 9 from Dataset 8 above.
